# Supplementary material for: Sound suppresses earliest visual cortical processing after sight recovery in congenitally blind humans
Source: Commun Biol. 2024 Jan 22;7:118. doi: 10.1038/s42003-023-05749-3 (PMC10803735; doi:10.1038/s42003-023-05749-3)
Supplement: Supplementary file 2 — Supplementary Information [file 42003_2023_5749_MOESM2_ESM.pdf]

Supplementary Information

Sound Suppresses Earliest Visual Cortical Processing After Sight Recovery in Congenitally Blind Humans

Suddha Sourav; Ramesh Kekunnaya; Davide Bottari; Idris Shareef; Kabilan Pitchaimuthu; Brigitte Röder

Supplementary Note 1: Complete Stimulus Table With Remapped Conditions

In Supp. Table 1, we provide the complete stimulus table for an experimental block, which, after remapping of electrode positions, led to the ERPs used for calculating the C1 difference wave ($\Delta C1$) values. For each stimulus type, the corresponding visual and auditory stimulus are shown. The rows marked with light orange were remapped (See *Methods*). Auditory stimuli, not reported in the present study, are marked in grey. Blue rows indicate the deviant (target) stimuli which underwent reaction time analysis for race model inequality (RMI) violation. We aimed for running 8 blocks per participant.

Supplementary Table 1: Complete stimulus table for the experiment.

Type	Visual Stim. Position	Auditory Stim. Pos	Trials/Block
Standard_visual	Top Left	None	8
Standard_visual	Top Right	None	8
Standard_visual	Bottom Left	None	8
Standard_visual	Bottom Right	None	8
Standard_auditory	None	Left	16
Standard_auditory	None	Right	16
Standard_bimodal	Top Left	Left	8
Standard_bimodal	Top Left	Right	8
Standard_bimodal	Top Right	Left	8
Standard_bimodal	Top Right	Right	8
Standard_bimodal	Bottom Left	Left	8
Standard_bimodal	Bottom Left	Right	8
Standard_bimodal	Bottom Right	Left	8
Standard_bimodal	Bottom Right	Right	8
Deviant_unimodal_v	Top Left	None	1
Deviant_unimodal_v	Top Right	None	1
Deviant_unimodal_v	Bottom Left	None	1
Deviant_unimodal_v	Bottom Right	None	1
Deviant_unimodal_a	None	Left	2
Deviant_unimodal_a	None	Right	2
Deviant_bimodal_v	Top Left	Left	1
Deviant_bimodal_v	Top Left	Right	1
Deviant_bimodal_v	Top Right	Left	1
Deviant_bimodal_v	Top Right	Right	1
Deviant_bimodal_v	Bottom Left	Left	1
Deviant_bimodal_v	Bottom Left	Right	1
Deviant_bimodal_v	Bottom Right	Left	1
Deviant_bimodal_v	Bottom Right	Right	1

Deviant_bimodal_a	Top Left	Left	1
Deviant_bimodal_a	Top Left	Right	1
Deviant_bimodal_a	Top Right	Left	1
Deviant_bimodal_a	Top Right	Right	1
Deviant_bimodal_a	Bottom Left	Left	1
Deviant_bimodal_a	Bottom Left	Right	1
Deviant_bimodal_a	Bottom Right	Left	1
Deviant_bimodal_a	Bottom Right	Right	1
Deviant_bimodal_av	Top Left	Left	1
Deviant_bimodal_av	Top Left	Right	1
Deviant_bimodal_av	Top Right	Left	1
Deviant_bimodal_av	Top Right	Right	1
Deviant_bimodal_av	Bottom Left	Left	1
Deviant_bimodal_av	Bottom Left	Right	1
Deviant_bimodal_av	Bottom Right	Left	1
Deviant_bimodal_av	Bottom Right	Right	1

Supplementary Note 2: Pilot Study

Research Question

The pilot experiment served to design the visual stimuli to lead to reliable $\Delta C1$ waves, and to formulate hypotheses about the following main experiment. The participants for the pilot study were tested in 2015. The analyses reported here are exploratory in nature.

Methods

Participants

Twenty-one typically sighted participants were tested. Due to a design error we had to discard the data of two participants, leaving 19 participants whose data were analyzed. Out of them, 13 were female, and 1 was left-handed (mean age: 27.89 years, range = 20 – 35 years). All participants were recruited from the local community of Hamburg, Germany, and provided written informed consent. The participants were free of any neurological disorders and had normal or corrected-to-normal vision.

Stimuli

The stimuli, with the following exceptions, were the same as in the actual study:

1. Participants sat in a relatively brightly lit room.

2. There were no auditory deviant stimuli, thus, all targets, both unimodal and bimodal, were single targets, where the sole deviant stimulus was visual.

3. A mean interstimulus interval of 1.1 s (uniform distributed, range: 1.0 – 1.2 s) was used.

Preprocessing and Data analysis

For calculating the $\Delta C1$, the preprocessing was essentially the same as reported in the method section of the main study. To investigate the presence of a $\Delta C1$, we first employed a cluster based permutation method for each condition (V, AV_i, and AV_c) separately, a method which we used in a previous study[1]. Here we also report the results of a Bayesian analysis.

Results

Qualitative Aspects

We observed similar C1 difference waves ($\Delta C1$) for the unimodal and the audiovisual conditions, with almost complete overlap (Supp. Fig. 1). Likewise, the topographies were remarkably similar across the three conditions (Supp. Fig. 2). The $\Delta C1$ peaks were however modest, generally ~ 1 μV at peak, and short-lived (~ 30 ms).

Quantitative Aspects

A cluster-based permutation test indicated the presence of a $\Delta C1$ in all three conditions (Supp. Fig. 3). Using a Bayesian hierarchical model on the average topography in the 50 – 100 ms time window for the $\Delta C1$, we found substantial evidence of a $\Delta C1$ in the *V* and the AV_c condition ($BF_{+0} > 3$ at electrodes O1, O2, P4, and Pz for condition *V*, and at P4 and Pz for condition AV_c). The evidence for AV_i was almost substantial (BF_{+0} = 2.96 at electrode Pz), but not above the limit of 3. Examination of the topographical plots indicated that at the last phase of the time window (90 – 100 ms), there was a zero crossing and associated topography change in all conditions, (Supp. Fig. 2), likely reducing the average $\Delta C1$ further. No significant differences between the $\Delta C1$ values emerged ($BF_{10} < 1.1$).

Supplementary Fig. 1: Time course of the $\Delta C1$ in the pilot experiment.

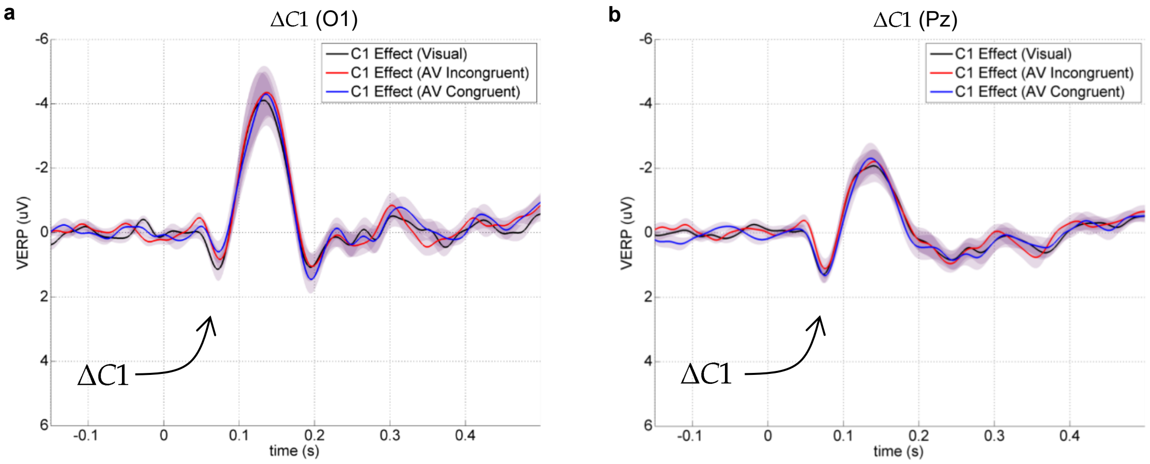

a. Time courses of the C1 effect ($\Delta C1$) for the visual and the audiovisual stimulus conditions in the pilot experiment (*N* = 19) at electrode O1. **b.** $\Delta C1$ time courses at the electrode Pz. Note the very similar time courses. Black lines: the visual (*V*) condition, red lines: the audiovisual incongruent (AV_i) condition, blue lines: the audiovisual congruent (AV_c) condition. Error bands represent the standard error of the mean.

Supplementary Fig. 2: Topography of the $\Delta C1$ in the pilot experiment.

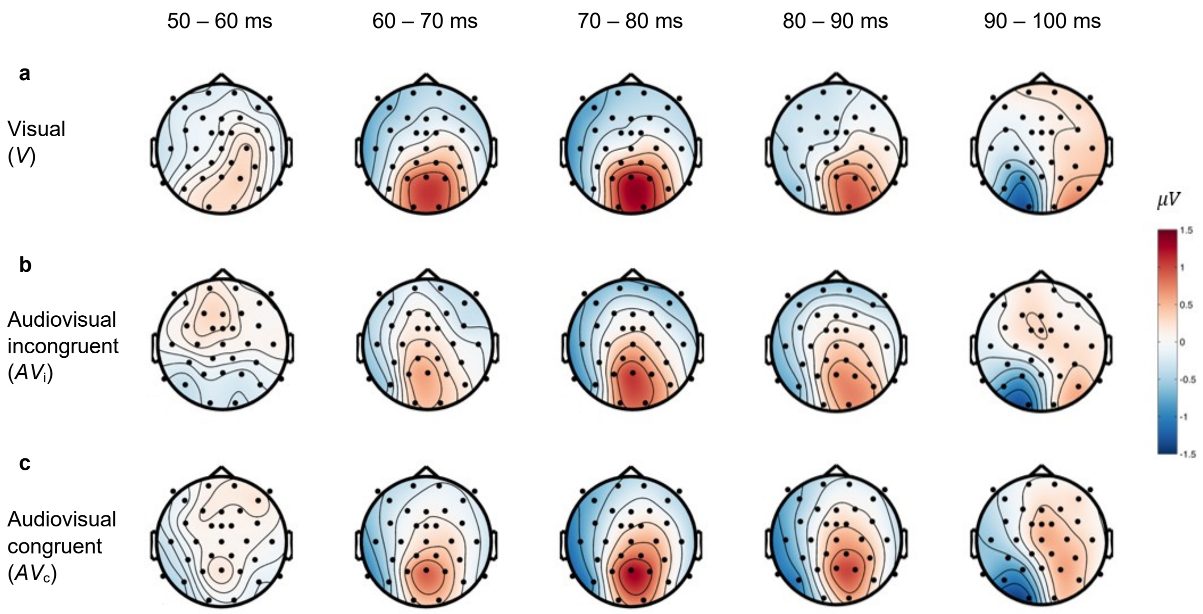

a. Topography of the $\Delta C1$ in the pilot experiment (N = 19), in steps of 10 ms, in the time range of 50 – 100 ms, for the unimodal visual (V) stimulus condition. **b.** and **c.** $\Delta C1$ topographies respectively for the audiovisual incongruent (AV_i) and audiovisual congruent (AV_c) stimulus conditions. Remarkably similar topographies were observed in all three conditions.

Supplementary Fig. 3: Cluster-based permutation test for ascertaining the presence of a $\Delta C1$ in the pilot experiment.

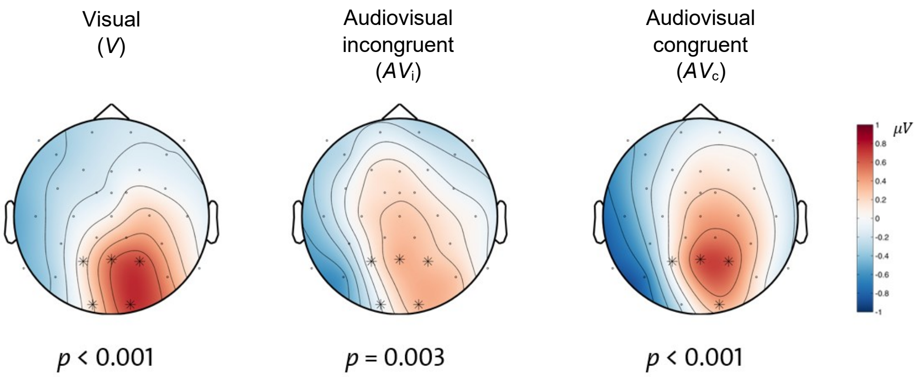

Results of a within-subject cluster-based permutation test, comparing the lower visual field (LVF) to the upper visual field (UVF) stimuli to ascertain the presence of a $\Delta C1$. A weighted cluster mean statistic from 10,000 draws with a one-sided t-test was used. Asterisks (*) indicate a statistically significant $\Delta C1$, with the p-values corrected for multiple comparison at the cluster level.

Design/Analysis Decisions for Following Studies

Based on the pilot study, we improved the subsequent experiment and data analysis in two ways. The first improvement was driven by the observation of relatively small $\Delta C1$ amplitudes in the pilot study. We reasoned that larger $\Delta C1$ values would increase statistical power, aiding the detection of possible $\Delta C1$ modulations. Based on the results, we took the following steps:

1. In subsequent studies[1,2], we chose to seat participants in a dimly (instead of brightly) lit room.

2. We increased the interstimulus interval to 1.85 s (uniform distributed, range: 1.5 – 2.2 s)

We observed larger $\Delta C1$ waves (~ 2 μV) on average in a following study[1], an observation which was replicated in the main experiment reported in the present article.

The second improvement stemmed from the fact that the present study had an obligate between-subject factor (*Group*), making the design inherently lower-powered. Testing a 3 × 2 interaction with a between subjects factor for $\Delta C1$ would have been prohibitively lower powered, with not enough CC/DC individuals in any global cohort to the best of our knowledge to reach 80% power[3]. The problem of a precipitous fall of power for detecting interactions, as a result of introducing a between-subject factor, has been termed "no-way interactions"[3], which likely would have plagued the present study without a more powerful statistical analysis strategy. However, many of the possible pairwise contrasts were of no immediate interest (see *Method*), and hierarchical (multilevel) models with partial pooling could estimate the contrasts of interest in a single model simultaneously. Therefore, we chose to test custom contrasts and adopted a Bayesian approach with a focus on estimation, allowing a more nuanced interpretation for potentially inconclusive results[4]. Moreover, Bayesian analysis is conservative at a single test level for small effect sizes, when applied with hierarchical (multilevel) models with weakly informative, non-flat priors as used in our study[5]. By this virtue it also does not require correction for multiple testings[5–7].

Supplementary Note 3: No Substantial Modulation of the $\Delta C1$ by Sounds in Sighted Controls

To test whether concurrent auditory stimulation modulated the $\Delta C1$ in typically sighted control individuals in the present study, we collapsed across the two sighted control groups (the MCC and MDC groups; *n* = 29, and ran the following planned comparisons simultaneously at the five electrodes (O1, O2, P3, P4, and Pz; see Supp. Fig. 4 for the cell means, i.e., the $\Delta C1$ amplitude estimates):

$$\Delta C1_{CONTROLS,V} - \Delta C1_{CONTROLS,AV_i}\ ;\ \Delta C1_{CONTROLS,V} - \Delta C1_{CONTROLS,AV_c}$$

Necessary orthogonal contrasts were added to make the contrast matrix full-rank[4].

We report the parameter estimates and the Bayes factors in Supp. Table 2. None of the differences were substantial or even anecdotal, in fact all tests of differences favored the null hypothesis.

Supplementary Fig. 4: The $\Delta C1$ over posterior electrodes (50 – 100 ms) in sighted controls.

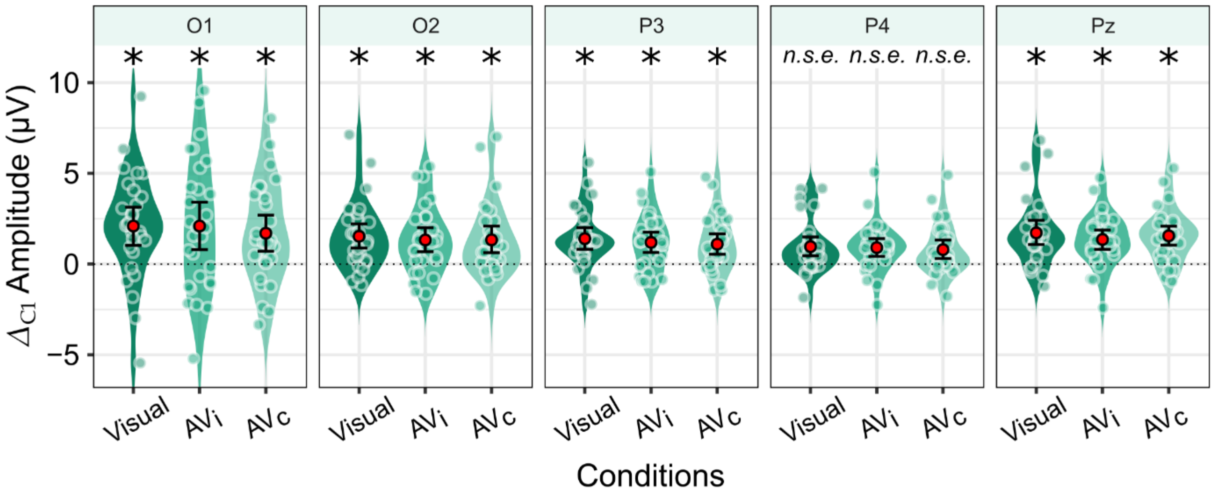

$\Delta C1$ amplitudes at the five posterior electrodes O1, O2, P3, P4, and Pz for the sighted control individuals (MCC + MDC, *n* = 29) in the visual as well as the audiovisual incongruent (AV_i) and audiovisual congruent (AV_c) stimulus

conditions. Asterisks (*) indicate substantial or stronger evidence for the presence of a $\Delta C1$ (BF_{+0} > 3) over the null hypothesis models, as well as positive region of practical equivalence (ROPE) tests, *n.s.e.* indicate no substantial evidence of a $\Delta C1$. Red circles indicate the mean values, and the error bars represent 95% confidence intervals for the means obtained by smoothed bootstrapping with Gaussian kernels. Individual data points have been jittered for readability.

Supplementary Table 2: Parameter estimates and Bayes factors for the custom hypotheses for testing $\Delta C1$ differences between the unimodal visual and the audiovisual conditions.

	Estimate	l-95% CI	u-95% CI	BF_{10}	ROPE %
Intercept	1.54	0.91	2.17	151.41*	0*
O1: $\Delta C1_{CONTROLS,V} - \Delta C1_{CONTROLS,AV_c}$	0.39	-0.34	1.12	0.125	26.69
O1: $\Delta C1_{CONTROLS,V} - \Delta C1_{CONTROLS,AV_i}$	-0.01	-0.73	0.72	0.072	43.95
O2: $\Delta C1_{CONTROLS,V} - \Delta C1_{CONTROLS,AV_c}$	0.19	-0.53	0.92	0.083	39.42
O2: $\Delta C1_{CONTROLS,V} - \Delta C1_{CONTROLS,AV_i}$	0.20	-0.53	0.92	0.084	38.61
P3: $\Delta C1_{CONTROLS,V} - \Delta C1_{CONTROLS,AV_c}$	0.30	-0.43	1.02	0.100	32.87
P3: $\Delta C1_{CONTROLS,V} - \Delta C1_{CONTROLS,AV_i}$	0.20	-0.53	0.92	0.084	38.71
P4: $\Delta C1_{CONTROLS,V} - \Delta C1_{CONTROLS,AV_c}$	0.16	-0.56	0.88	0.079	40.71
P4: $\Delta C1_{CONTROLS,V} - \Delta C1_{CONTROLS,AV_i}$	0.05	-0.68	0.77	0.073	43.85
Pz: $\Delta C1_{CONTROLS,V} - \Delta C1_{CONTROLS,AV_c}$	0.16	-0.56	0.89	0.079	40.47
Pz: $\Delta C1_{CONTROLS,V} - \Delta C1_{CONTROLS,AV_i}$	0.36	-0.36	1.09	0.119	28.54

Asterisks (*) indicate Bayes Factors indicating substantial evidence (BF_{10} > 3), or a positive *region of practical equivalence* (ROPE) test.

Supplementary Note 4: Analysis of Race Model Violation: A Non-Parametric Frequentist Approach

The Race Model Inequality

The race model inequality (RMI) puts an upper bound to the statistical facilitation obtained from combining two separate sensory modalities. For unimodal auditory target stimuli (*A*), unimodal visual target stimuli (*V*) and their combinations (*AV*), the reaction time t must obey the following inequality (for details, see Gondan and Minakata, 2016[8]):

$$P(t \leq \tau)_{AV} \leq \; P(t \leq \tau)_A + P(t \leq \tau)_V$$

Where the subscripts denote the stimulus condition. Using the notation for cumulative distribution functions, $P(t \leq \tau)_x = F_x(\tau)$ and a change of variable, the equation can be rewritten as:

$$F_{AV}(t) \leq F_A(t) + F_V(t) \quad \text{--- [1]}$$

A cumulative distribution function, e.g., $F_{AV}(t)$, has an upper bound of 1. Therefore, the right side of inequality [1] can be rewritten as

$$F_{AV}(t) \leq \min\,[F_A\;\; t) + F_V\;\; t), 1]$$

or equivalently, $F_{AV}(t) - \min\,[F_A(t) + F_V(t), 1] \leq \; 0$ -------------------- [2]

The left-hand side of inequality [2] is the race model violation function. In nonparametric frequentist approaches[8,9], significant departures of race model violation at any time point (or reaction time percentile) is investigated.

Target Stimulus Table

Below, we list all target stimulus types for subsequent RMI violation testing. We were not interested in running RMI violation tests for separate quadrants or stimulation sides, but rather in investigating the effect of the spatial congruence of the audiovisual stimuli, and their general stimulus energy (i.e., unimodal vs. bimodal stimuli). For denoting the target stimuli, we employed a notation ignoring the visual stimulus quadrants, as outlined in Supp. Table 3.

Supplementary Table 3: Target stimulus notation for investigating race model violations.

Notation	Auditory Stimulus	Visual Stimulus
A_L^T	Left, Target	–
A_R^T	Right, Target	–
V_L^T	–	Left, Target
V_R^T	–	Right, Target
$A_L^T V_L^0$	Left, Target	Left, Standard
$A_L^T V_R^0$	Left, Target	Right, Standard
$A_R^T V_L^0$	Right, Target	Left, Standard
$A_R^T V_R^0$	Right, Target	Right, Standard
$A_L^0 V_L^T$	Left, Standard	Left, Target
$A_L^0 V_R^T$	Left, Standard	Right, Target
$A_R^0 V_L^T$	Right, Standard	Left, Target
$A_R^0 V_R^T$	Right, Standard	Right, Target
$A_L^T V_L^T$	Left, Target	Left, Target
$A_L^T V_R^T$	Left, Target	Right, Target
$A_R^T V_L^T$	Right, Target	Left, Target
$A_R^T V_R^T$	Right, Target	Right, Target

Tested Inequalities

We aimed to answer two questions and checked for RMI violations with two corresponding groups of inequalities. First, we were interested in finding if bimodal double targets ($A^T V^T$) were faster than bimodal single targets ($A^T V^0, A^0 V^T$).

1. For congruent stimuli:

$$F\left(\left\{A_L^T V_L^T, A_R^T V_R^T\right\}\right) \leq F\left(\left\{A_L^T V_L^0, A_R^T V_R^0\right\}\right) + F\left(\left\{A_L^0 V_L^T, A_R^0 V_R^T\right\}\right)$$

2. For incongruent stimuli:

$$F\left(\left\{A_L^T V_R^T, A_R^T V_L^T\right\}\right) \leq F\left(\left\{A_L^T V_R^0, A_R^T V_L^0\right\}\right) + F\left(\left\{A_L^0 V_R^T, A_R^0 V_L^T\right\}\right)$$

Here, {} indicates that we collapsed across the sides.

Second, we investigated if bimodal target stimuli ($A^T V^T$) were faster than unimodal target stimuli (A^T, V^T). The inequalities tested were:

3. For congruent stimuli:

$$F\left(\left\{A_L^T V_L^T, A_R^T V_R^T\right\}\right) \leq F\left(\left\{A_L^T, A_R^T\right\}\right) + F\left(\left\{V_L^T, V_R^T\right\}\right)$$

4. For incongruent stimuli:

$$F\left(\left\{A_L^T V_R^T, A_R^T V_L^T\right\}\right) \leq F\left(\left\{A_L^T, A_R^T\right\}\right) + F\left(\left\{V_R^T, V_L^T\right\}\right)$$

Note that all terms in inequalities 1 and 2 have a visual and an auditory stimulus appearing together. While these stimuli were not identical (see *Methods*), the stimulus energies were comparable. In inequalities 3 and 4, bimodal and unimodal stimuli are compared, and the energies across the stimuli are not similar.

Further Details on Reaction Time Data of Participants

The data of 1 CC participant and 2 DC participants who could not reliably discriminate the grating orientations and thus observed the stimuli passively, and the data of an additional CC participant for whom experimenter response coding was used, were excluded from the reaction time analyses along with their matched control participants (N_{CC} = N_{MCC} = 12; N_{DC} = N_{MDC} = *13).*

For investigating RMI violations, we followed the guidelines of Gondan et al.[8,9], examining the race model with a cluster based permutation test with 10,001 permutation on the 5th – 30th percentiles in steps of 5 percentiles. The t_{sum} statistic was used for permutation. As we tested multiple conditions and had no a-priori hypotheses, we corrected the cluster-based permutation tests' *p*-values further with a Benjamini-Hochberg procedure in R, version 4.2.2[10].

Since participants responded with a computer mouse, we verified that the mouse response latency was relatively constant across the stimuli (mean delay: 19 – 20 ms, standard deviation of delay < 3 ms in all conditions). Thus, the race model inequality cannot be explained through highly variable but systematically different reaction times across conditions driven by the mouse response latency.

Results

The reaction time benefit conferred by bimodal double target stimuli ($A^T V^T$), compared to bimodal targets where only the auditory ($A^T V^0$), or only the visual stimulus ($A^0 V^T$) was deviant, served to test the RMI inequality when similar stimulus energy was delivered to both the auditory and the visual modality across the conditions (Supp. Fig. 5a). In this comparison, we observed evidence for audiovisual integration above the level predicted by chance, in each group and condition regardless of spatial congruence (all *p*s < 0.05, cluster-based permutation tests followed by Benjamini-Hochberg correction for multiple comparisons). Additionally, we found no substantial evidence that the positive RMI violation area, a marker of the amount of RMI violation[11], was different for groups and/or spatial congruence (Bayesian linear mixed model, BF_{10} for intercept: CC/MCC: 633.35, DC/MDC: 26.39, for all other factors e.g. *Group*, *Congruence*, and *Group* × *Congruence*, BF_{10} < 1).

Supplementary Fig. 5: Audiovisual integration after sight recovery.

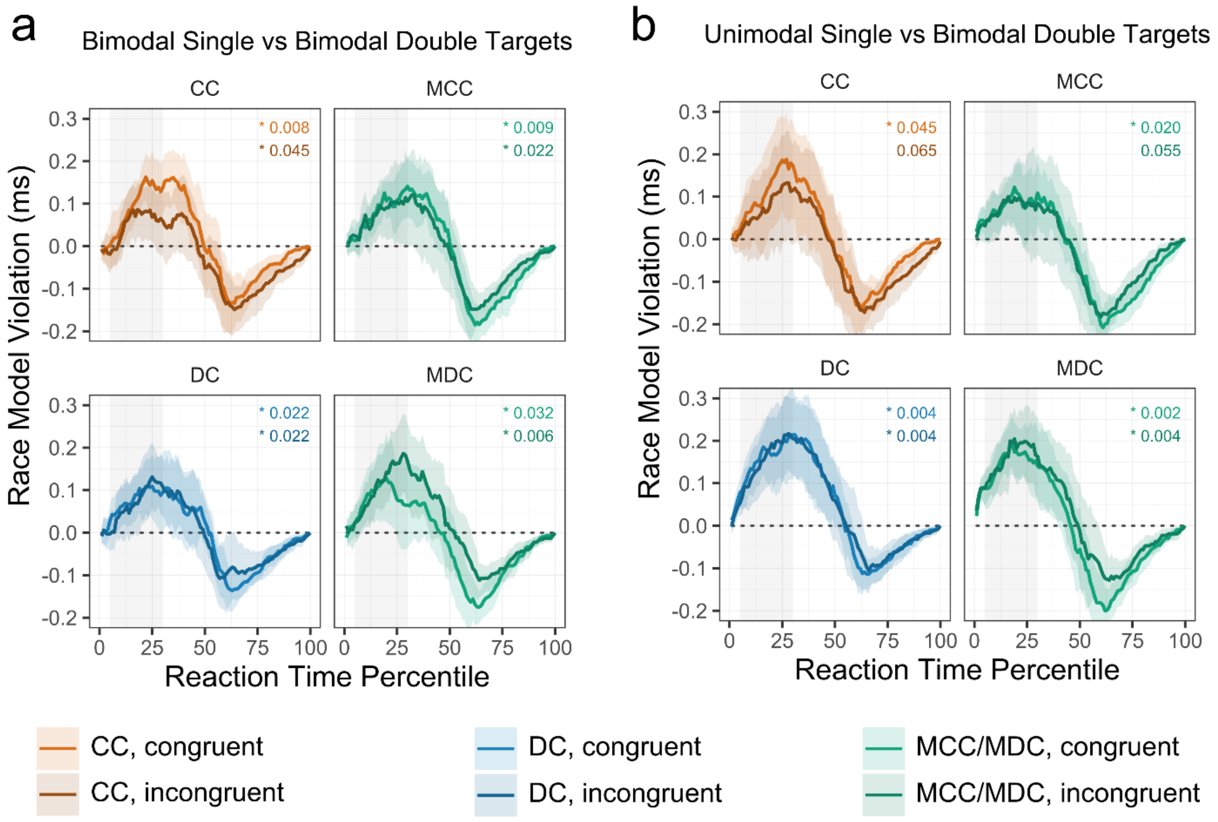

Plots indicating the amount of race model inequality (RMI) violation (in ms) as a function of reaction time percentiles for all participant groups and conditions, with positive values indicating an audiovisual integration beyond that expected by chance alone. The grey area marks the region for testing the race model inequality (5th – 30th percentiles in steps of 5)[9]. **a.** RMI violations for reaction times from bimodal (*AV*) targets, where either one (A^TV^0/A^0V^T) or both stimuli (A^TV^T) were targets. Participants reacted faster when both stimuli were targets, and consistent evidence of audiovisual integration was found in all groups and conditions. The positive violation area under the curves did not substantially differ between groups or conditions (Bayesian mixed models). **b.** RMI violations for reactions times to bimodal double targets (A^TV^T) vs. unimodal single (A^T or V^T) targets. Unlike in **a.**, this comparison does not compare combinations with similar stimulus energy in both modalities. We found significant audiovisual integration in most conditions and groups except in the CC and the MCC groups in the incongruent target conditions. No substantial evidence for a difference between groups or conditions were, however, detected. (*: $p < 0.05$, *p*-values from cluster-based permutation in the RMI test region, thereafter corrected with the Benjamini-Hochberg procedure. Shaded area indicates the 95% confidence interval obtained with smoothed bootstraps with Gaussian kernels[12]).

We additionally investigated the RMI violation for bimodal double target stimuli (A^TV^T) compared to the unimodal targets (A^T or V^T, Supp. Fig. 5b). This analysis, unlike the analysis including only bimodal targets, does not compare stimuli with equal energy across the modalities[8]. For this analysis, significant RMI violations emerged except in the spatially incongruent combinations for both the CC and the MCC groups. No substantial group- and/or congruence-based differences for the violation areas were found with the Bayesian mixed models (BF_{10} for intercept = CC/MCC: 975.70, DC/MDC: 626.49, all other factors i.e., Group, Congruence, and Group × Congruence, BF_{10} < 1).

The non-parametric frequentist approach confirmed the Bayesian approach reported in the article with a temporally finer-grained analysis.

Supplementary Note 5: Participant Characteristics

Here, in Supp. Tables 4 and 5, we respectively provide the characteristics of the congenital cataract reversal (CC) and developmental cataract reversal (DC) participants. Please note that the tables are based on Sourav et al. (2020), *Experiment 2*[2].

Supplementary Table 4: Participant characteristics for the sight recovery individuals with a history of congenital bilateral dense cataracts and subsequent surgery (CC).

Participant ID	Group	Age (yrs.)	Duration of blindness (mo.)	Time Since Surgery (mo.)	Sex	Handedness	Visual Acuity (Decimal)	Visual Acuity Prior to Surgery (Better Eye)	Nystagmus	Strabismus	Family History
CC – 001	CC	26	5	308	M	Right	0.180	NA	Yes	Esotropia	No
CC – 005	CC	33	72	330	M	Right	0.051	NA	Yes	No	Yes
CC – 014	CC	6	5	70	M	Right	0.240	Not fixating/following light	Yes	No	No
CC – 002	CC	39	24	444	M	Right	0.400	NA	Yes	Esotropia	Yes
CC – 004	CC	14	4	175	M	Right	0.130	Fixates and follows light	Yes	Exotropia	No
CC – 006	CC	24	4	294	M	Left	0.130	NA	Yes	Esotropia	Yes
CC – 007	CC	15	15	166	M	Right	0.500	Fixates and follows light	Yes	Exotropia	Yes
CC – 008	CC	9	1	116	M	Right	0.700	Fixates and follows light	No	No	Yes
CC – 009	CC	11	11	129	M	Right	0.240	Fixates and follows light	Yes	Esotropia	No
CC – 015	CC	8	48	48	F	Right	0.150	Perception of light	Yes	No	Yes
CC – 013	CC	11	74	61	M	Right	0.250	0.017*	Yes	No	Yes
CC – 012	CC	11	42	90	F	Right	0.450	Fixates and follows light	Yes	Exotropia	Yes
CC – 011	CC	21	213	40	M	Right	0.200	Counting fingers at 0.5 m	Yes	Esotropia	Yes
CC – 016	CC	11	72	68	F	Right	0.260	Fixates and follows light	Yes	No	No

* CC – 013 had absorbed cataracts at presentation

Supplementary Table 5: Participant characteristics for the sight recovery individuals with a history of developmental bilateral cataracts and subsequent surgery (DC).

Participant ID	Group	Age	Age at Surgery (yrs.)	Time Since Surgery (mo.)	Sex	Handedness	Visual Acuity (Decimal)	Visual Acuity Prior to Surgery (Better Eye)	Nystagmus	Strabismus	Family History
DC – 002	DC	19	14	55	M	Right	1.000	0.200	No	No	No
DC – 011	DC	14	7	80	F	Right	0.530	Finger counting at 1 m	No	Exotropia	Yes
DC – 003	DC	13	6	74	F	Right	1.000	0.500	No	No	No
DC – 005	DC	14	8	80	M	Right	0.710	0.100	No	Exotropia	No
DC – 007	DC	12	6	77	M	Right	1.000	0.250	No	No	Yes
DC – 014	DC	12	7	66	M	Right	0.230	0.125	No	No	No
DC – 015	DC	12	7	67	M	Right	0.460	0.100	No	No	No
DC – 009	DC	16	10	67	M	Right	0.740	0.400	No	No	No
DC – 010	DC	24	2	264	F	Right	0.440	Fixates and follows light	No	Exotropia	No
DC – 008	DC	18	12	74	F	Right	0.790	Perceives hand movement	No	No	No
DC – 016	DC	17	12	60	M	Right	0.310	0.160	No	No	Yes
DC – 017	DC	9	2	86	M	Right	0.930	0.154	No	No	No
DC – 018	DC	16	6	113	M	Right	0.520	Finger counting at 1 m	No	No	No
DC – 019	DC	10	3	76	M	Left	0.730	0.065	No	No	Yes
DC – 020	DC	11	1	115	M	Right	0.667	0.154	No	No	No

Supplementary Note 6: C1 Wave Time Courses for Upper and Lower Visual Field Stimulation

We provide the C1 waves for the upper vs. lower visual field stimulations from the main experiment below (UVF/LVF, Supp. Fig. 6). Subtracting the UVF from the LVF stimulation condition derives the $\Delta C1$ (see Fig. 1 and Fig. 2e in the article).

There are two potential confounds related to interpreting separate C1 wave time courses which we briefly outline here. The first confound is that for the audiovisual conditions (AV_i and AV_c), the visual event-related activity is accompanied with concurrent auditory event-related activity which is often larger on the scalp than the C1 wave itself. This confound would have made interpretation of the UVF vs. LVF waves difficult if not impossible, masking the visual activity to a large extent. To sidestep this confound, for the audiovisual conditions we subtracted the unimodal auditory event-related potential (AERP) for plotting. For the audiovisual congruent conditions (AV_c), to derive the AERP, the electrodes in all EEG epochs with unimodal left auditory field stimulation were remapped and averaged together with the EEG epochs generated from unimodal right auditory field stimulation, as for the visual stimulation (see *Method*), serving as an estimate for the ipsilateral (right) auditory stimulus related activity. Thus, the plots depict ($AV_{c,\ UVF} - A_R$) and ($AV_{c,\ LVF} - A_R$) instead of ERPs to $AV_{c,\ UVF}$ and $AV_{c,\ LVF}$. Similarly, for plotting the C1 waves in the audiovisual incongruent conditions (AV_i), we subtracted the AERP estimate of the contralateral auditory stimulation (A_L), derived by remapping the electrodes of all unimodal right auditory field stimulation condition before averaging them together with EEG epochs generated from the left visual field stimulation.

Supplementary Fig. 6: Time courses for the C1 waves at electrode O1 for upper visual field (UVF) and lower visual field (LVF) stimulation in all groups (CC/MCC/DC/MDC) and stimulus conditions.

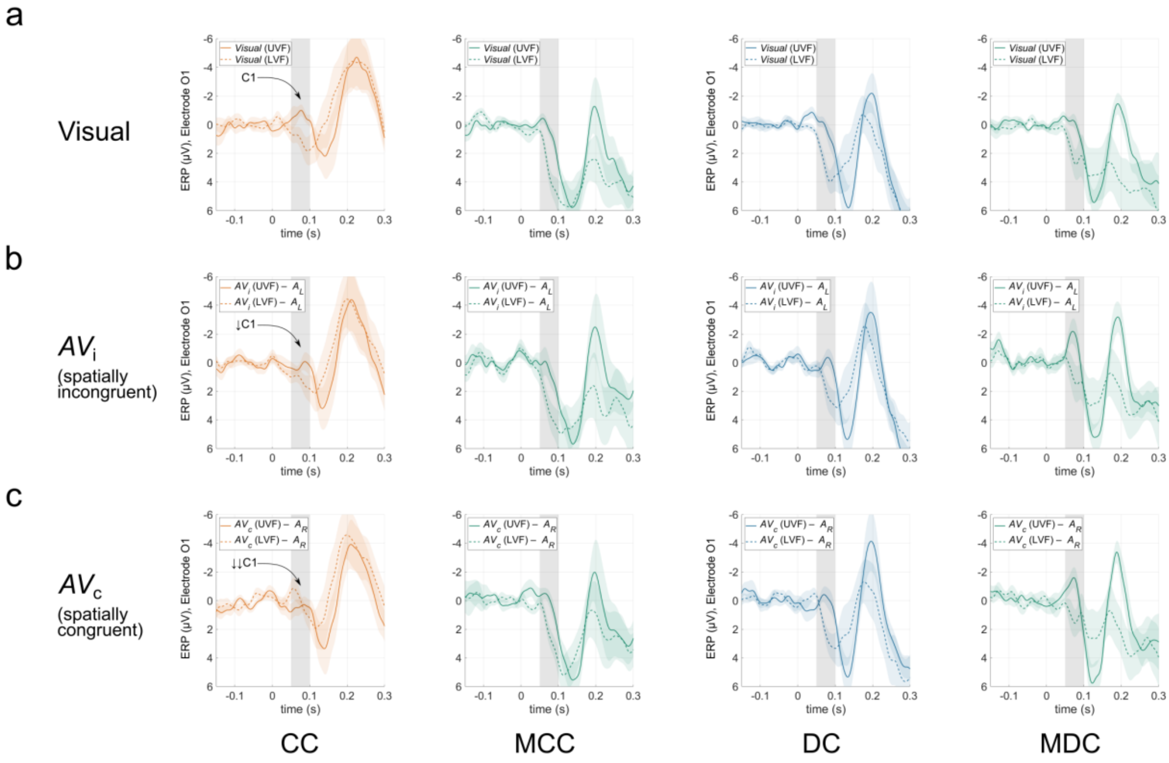

a. C1 waves for the unimodal visual (*V*) stimulation condition. **b.** C1 waves for the spatially incongruent audiovisual stimulation condition (AV_i), with the unimodal auditory event-related potential subtracted for interpretation. **c.** C1 waves for the spatially congruent audiovisual stimulation condition (AV_c), with the unimodal auditory event-related potential subtracted for interpretation. Error bands represent the standard error of the mean. The grey bar indicates the 50 – 100 ms range employed in the analyses.

The second confound is that early visual or audiovisual non-retinotopic activity is not accounted for in the separate C1 wave plots. Early non-retinotopic visual activity (e.g., the early P1 wave) onset have been reported to be only 10-20 ms after C1 wave onsets, making the calculation of $\Delta C1$ necessary for investigating feedforward retinotopic visual activity[13]. Moreover, overlapping non-retinotopic audiovisual activity might lead to an overall shift of both waves for the audiovisual conditions.

Despite these caveats, the pattern of activity captured by the $\Delta C1$ was faithfully reflected in the separate C1 wave plots for UVF vs. LVF stimulation as well: In all groups and conditions, we observed C1 waves with canonical appearance except in the CC group for the audiovisual conditions. For the AV_i condition, partially overlapping C1 waves were qualitatively observed; for the AV_c condition, we observed an initial reversal of the expected C1 direction followed by overlapping C1 waves for UVF vs. LVF conditions.

Supplementary References

1. Sourav, S., Bottari, D., Kekunnaya, R. & Röder, B. Evidence of a retinotopic organization of early visual cortex but impaired extrastriate processing in sight recovery individuals. *Journal of Vision* **18**, 22 (2018).
2. Sourav, S., Bottari, D., Shareef, I., Kekunnaya, R. & Röder, B. An electrophysiological biomarker for the classification of cataract-reversal patients: A case-control study. *EClinicalMedicine* **27**, 100559 (2020).
3. Simonsohn, U. [17] No-way Interactions. *The Winnower* (2015). doi:10.15200/winn.142559.90552.
4. Schad, D. J., Vasishth, S., Hohenstein, S. & Kliegl, R. How to capitalize on a priori contrasts in linear (mixed) models: A tutorial. *Journal of Memory and Language* **110**, 104038 (2020).
5. Gelman, A. & Tuerlinckx, F. Type S error rates for classical and Bayesian single and multiple comparison procedures. *Computational Statistics* **15**, 373–390 (2000).
6. Gelman, A., Hill, J. & Yajima, M. Why We (Usually) Don’t Have to Worry About Multiple Comparisons. *Journal of Research on Educational Effectiveness* **5**, 189–211 (2012).
7. Limbachia, C. *et al.* Controllability over stressor decreases responses in key threat-related brain areas. *Commun Biol* **4**, 1–11 (2021).
8. Gondan, M. & Minakata, K. A tutorial on testing the race model inequality. *Attention, Perception, & Psychophysics* **78**, 723–735 (2016).
9. Gondan, M. A permutation test for the race model inequality. *Behavior Research Methods* **42**, 23–28 (2010).

10. R Core Team. R: A language and environment for statistical computing. (2016). https://www.r-project.org/

11. Colonius, H. & Diederich, A. The race model inequality: Interpreting a geometric measure of the amount of violation. *Psychological Review* **113**, 148–154 (2006).

12. Wołodźko, T. Kernelboot: Smoothed bootstrap and random generation from kernel densities. (2019). https://cran.r-project.org/package=kernelboot

13. Qu, Z. & Ding, Y. Identifying and removing overlaps from adjacent components is important in investigations of C1 modulation by attention. *Cognitive Neuroscience* **9**, 64–66 (2018).
