# Supplementary material for: Sound suppresses earliest visual cortical processing after sight recovery in congenitally blind humans
Source: Commun Biol. 2024 Jan 22;7:118. doi: 10.1038/s42003-023-05749-3 (PMC10803735; doi:10.1038/s42003-023-05749-3)
Supplement: Supplementary file 3 — Description of Additional Supplementary Files [file 42003_2023_5749_MOESM3_ESM.pdf]

Description of Additional Supplementary Files

File Name: Supplementary Data 1
Description: The source data behind the graphs in Fig. 2d in the paper

File Name: Supplementary Data 2
Description: The source data behind the graphs in Fig. 4a in the paper

File Name: Supplementary Data 3
Description: The source data behind the graphs in Fig. 4b in the paper

File Name: Supplementary Data 4
Description: The source data behind the graphs in Fig. 4c in the paper
