# Supplementary material for: Sound suppresses earliest visual cortical processing after sight recovery in congenitally blind humans
Source: Commun Biol. 2024 Jan 22;7:118. doi: 10.1038/s42003-023-05749-3 (PMC10803735; doi:10.1038/s42003-023-05749-3)
Supplement: Supplementary file 8 — Reporting Summary [file 42003_2023_5749_MOESM8_ESM.pdf]

nature portfolio

Corresponding author(s): Dr. Suddha Sourav

Last updated by author(s): Dec 21, 2023

Reporting Summary

Nature Portfolio wishes to improve the reproducibility of the work that we publish. This form provides structure for consistency and transparency in reporting. For further information on Nature Portfolio policies, see our Editorial Policies and the Editorial Policy Checklist.

Statistics

For all statistical analyses, confirm that the following items are present in the figure legend, table legend, main text, or Methods section.

n/a	Confirmed	
☐	☒	The exact sample size (*n*) for each experimental group/condition, given as a discrete number and unit of measurement
☐	☒	A statement on whether measurements were taken from distinct samples or whether the same sample was measured repeatedly
☐	☒	The statistical test(s) used AND whether they are one- or two-sided<br>*Only common tests should be described solely by name; describe more complex techniques in the Methods section.*
☒	☐	A description of all covariates tested
☐	☒	A description of any assumptions or corrections, such as tests of normality and adjustment for multiple comparisons
☐	☒	A full description of the statistical parameters including central tendency (e.g. means) or other basic estimates (e.g. regression coefficient) AND variation (e.g. standard deviation) or associated estimates of uncertainty (e.g. confidence intervals)
☐	☒	For null hypothesis testing, the test statistic (e.g. *F*, *t*, *r*) with confidence intervals, effect sizes, degrees of freedom and *P* value noted<br>*Give P values as exact values whenever suitable.*
☐	☒	For Bayesian analysis, information on the choice of priors and Markov chain Monte Carlo settings
☐	☒	For hierarchical and complex designs, identification of the appropriate level for tests and full reporting of outcomes
☐	☒	Estimates of effect sizes (e.g. Cohen's *d*, Pearson's *r*), indicating how they were calculated

Our web collection on statistics for biologists contains articles on many of the points above.

Software and code

Policy information about availability of computer code

Data collection	Stimulus presentation: PsychoPy (v.1.83);<br>EEG data collection: BrainVision Recorder (v.1.20)
Data analysis	EEG Preprocessing: Matlab (v.2012b), EEGLAB (v.11.5.4b);<br>R Analysis: R (v.4.2.2), attached packages_versions: effsize_0.8.1, MASS_7.3-58.3, bayesplot_1.10.0, kernelboot_0.1.9, bayestestR_0.13.0, sjmisc_2.8.9, sjstats_0.18.2, brms_2.19.0, Rcpp_1.0.10, readxl_1.4.2, nlme_3.1-162<br>R.matlab_3.7.0, ggplot2_3.4.2, tidyr_1.3.0, dplyr_1.1.2<br>Source Analysis: sLORETA implemented in Brainstorm (v.15-Aug-2023), running on Matlab (v.R2022a)<br><br>Custom code has been uploaded at https://doi.org/10.25592/uhhfdm.13468

For manuscripts utilizing custom algorithms or software that are central to the research but not yet described in published literature, software must be made available to editors and reviewers. We strongly encourage code deposition in a community repository (e.g. GitHub). See the Nature Portfolio guidelines for submitting code & software for further information.

Data

Policy information about availability of data

All manuscripts must include a data availability statement. This statement should provide the following information, where applicable:

- Accession codes, unique identifiers, or web links for publicly available datasets
- A description of any restrictions on data availability
- For clinical datasets or third party data, please ensure that the statement adheres to our policy

Aggregated, pseudonymized data as well as software code required to replicate the results have been deposited at the University of Hamburg research data repository (https://doi.org/10.25592/uhhfdm.13468). These data will be made available to external investigators upon reasonable request to the corresponding author through data transfer agreements approved by the stakeholders, under stipulations of applicable law including but not limited to the General Data Protection Regulation (GDPR; EU 2016/679).

Human research participants

Policy information about studies involving human research participants and Sex and Gender in Research.

Reporting on sex and gender	The present study employed a mixed design involving within-subject conditions as well as groups of sight recovery individuals and their typically sighted matched control participants. Custom contrasts quantified between-group as well as within-group differences. Crucially, each sight recovery participant was matched to a typically sighted control individual based on age, sex, and handedness (see Methods: Participants). All participants self-reported sex; in addition the sight-recovery participants' medical reports were consistent with their self reports.
Population characteristics	See the Behavioural & Social Sciences Study Design section.
Recruitment	Both the congenital and developmental cataract reversal individuals were tested at the L V Prasad Eye Institute (LVPEI), Hyderabad, India; control participants were recruited from the local community of Hamburg, Germany. We specifically used a group of sight restored developmental cataract individuals to rule out potential biases including surgery-related factors (e.g., seeing with intraocular lenses) and the role of vision after birth. In addition, the inclusion of the developmental catarct reversal group prevents confounds based on testing site or participant ethnicity. The present pattern of results additionally cannot be explained by self-selection biases because the participants could not choose their group membership (see Methods and Results).
Ethics oversight	The study was jointly approved by the local ethical commission of the LVPEI and of the faculty of Psychology and Human Movement at University of Hamburg.

Note that full information on the approval of the study protocol must also be provided in the manuscript.

Field-specific reporting

Please select the one below that is the best fit for your research. If you are not sure, read the appropriate sections before making your selection.

☐ Life sciences ☒ Behavioural & social sciences ☐ Ecological, evolutionary & environmental sciences

For a reference copy of the document with all sections, see nature.com/documents/nr-reporting-summary-flat.pdf

Behavioural & social sciences study design

All studies must disclose on these points even when the disclosure is negative.

Study description	Quantitative, experimental, cross-sectional design
Research sample	The study sample was chosen because of their importance in elucidating the role of early developmental vision. Fourteen sight recovered participants with a history of total, dense, and bilateral congenital cataracts took part in the experiment (CC group, mean age = 17.07 years, range = 6 – 39 years). The CC participants were not able to perceive more than diffuse light through the cataracts before their surgery (mean duration of visual deprivation = 42.14 months, range = 1 month – 17.75 years). They were tested at least three years after undergoing surgery (see Method: Participants), guaranteeing an extended time for visual recovery (geometric mean visual acuity at test = 0.229, decimal units, range = 0.051 – 0.7). Fifteen additional participants with a history of bilateral developmental cataracts (DC group), who underwent the same surgical procedures, were included as a control group (mean age = 14.47 years, range = 9 – 24 years). This group was considered to control for surgery-related factors (e.g., seeing with intraocular lenses) and the role of vision after birth. For each CC and DC individual, a typically sighted participant matched for age, sex, and handedness took part (MCC: matched controls for the CC group, n = 14, MDC: Matched controls for the DC group, n = 15). The CC and DC groups were tested at the L V Prasad Eye Institute in Hyderabad, India, and the typically sighted control participants were tested in Hamburg, Germany (see Method: Participants). Sample size was based on testing feasibility in a rare group of well characterized sight recovery individuals, and previous studies reporting a similiar number of participants (~15). In addition we tested

	custom contrasts and adopted a Bayesian approach with a focus on estimation, allowing a more nuanced interpretation for potentially inconclusive results.
Sampling strategy	The sight recovery participants were recruited from the patient pool of the L V Prasad Eye Institute in Hyderabad, India.
Data collection	Electroencephalographic (EEG) data were collected from the participants as they performed a behavioral task (See Method). Data were continuously recorded with an EEG amplifier connected to a PC-based recording system. An experimenter and in some cases, a parent or another legal guardian was present in the room where the experiment was run, maintaining sufficient distance behind the participant. Experimental conditions were randomized, and each stimulus lasted 150 ms. The experimenters were not blind to the conditions or hypotheses.
Timing	Data collection start: 02/12/2015, data collection stop: 05/12/2019
Data exclusions	We excluded 2 participants from EEG analysis: one due to a lack of etiological certainty (i.e., congenital vs. developmental origins), and the other because of a history of neurological and developmental disorders. These exclusion-criteria were pre-established. The data of 1 CC participant and 2 DC participants who could not reliably discriminate the grating orientations and thus observed the stimuli passively, and the data of an additional CC participant for whom experimenter response coding was used, were excluded from the reaction time analyses along with their matched control participants (n_CC = n_MCC = 12; n_DC = n_MDC = 13).
Non-participation	None dropped out/declined participation.
Randomization	Participants were not allocated to the groups by the experimenters randomly. Rather, group membership was based on the participants' history of early visual experience (i.e., history of total dense bilateral congenital cataracts/bilateral developmental cataracts/typical visual history). The group diagnosis was performed by a panel involving ophthalmologists and optometrists based on medical records and clinical examinations.

Reporting for specific materials, systems and methods

We require information from authors about some types of materials, experimental systems and methods used in many studies. Here, indicate whether each material, system or method listed is relevant to your study. If you are not sure if a list item applies to your research, read the appropriate section before selecting a response.

Materials & experimental systems

n/a	Involved in the study
☒	☐ Antibodies
☒	☐ Eukaryotic cell lines
☒	☐ Palaeontology and archaeology
☒	☐ Animals and other organisms
☒	☐ Clinical data
☒	☐ Dual use research of concern

Methods

n/a	Involved in the study
☒	☐ ChIP-seq
☒	☐ Flow cytometry
☒	☐ MRI-based neuroimaging
